# Vascular screening in asymptomatic subjects using non-contrast MRA

**DOI:** 10.1186/1532-429X-14-S1-P129

**Published:** 2012-02-01

**Authors:** Yuki Sekine-Ohmoto, Makiko Ishihara, Takashi Yoshida, Junji Takahashi, Takashi Yamamoto, Hiroyuki Tsuji, Yasuji Arase

**Affiliations:** 1 Health Management Center, Toranomon Hospital, Tokyo, Japan; 2 Imaging Center, Toranomon Hospital, Tokyo, Japan; 3 Radiology, Toranomon Hospital, Tokyo, Japan

## Summary

Vascular screening including the coronary, carotid, renal arteries and aorta using non-contrast MRA in 300 asymptomatic subjects detected atherosclerosis lesions including the coronary artery in 5% of participants.

## Background

Atherosclerosis is a systemic disease affecting arteries in a whole body and a major cause of death in the world. There are few data, however, on the extent of the diseases associated with atherosclerosis in asymptomatic subjects.

Objective of this study was to identify the prevalence of cardiovascular diseases in asymptomatic subjects using non-contrast MRA.

## Methods

We performed vascular screening including the coronary, carotid, renal arteries and aorta using MRA (1.5 T commercial scanner, Toshiba Medical Systems Excelart Vantage TM powered by Atlas) and biochemistry tests in asymptomatic subjects from October 2008 to July 2011. Imaging of carotid arteries was performed by time of flight methods. Imaging of coronary arteries was performed by corrected respiratory-triggered and cardiac-triggered steady-state-free-precision (SSFP) sequence with fat suppression and T2 preparation. The data of the magnetic resonance coronary angiography (MRCA) was transferred to the workstation with image reconstruction software (AZE VirtualPlace Fujin, AZE Ltd., Tokyo, Japan). Imaging of renal arteries was performed by steady-state-free-precision (SSFP) sequence with time-SLIP pulse. The diseases of the aorta were detected by sagittal images of the coronary artery and coronal images of the renal artery by which the whole aorta from the ascending aorta to common iliac arteries was covered.

## Results

There were 186 males and 114 females with a mean age of 59±10 and 64±9, respectively. Six subjects (2%) had coronary diseases, (occluded in two, severe stenosis in four) six (2%) had carotid artery diseases (internal carotid artery occluded in two, moderate stenosis in four), two (0.7%) had renal artery disease, two had aortic disease (thoracic aortic aneurysm), and two had peripheral artery (common ileac artery) aneurysms. Two had atherosclerotic diseases in 2 lesions simultaneously. Thoracic aortic aneurysm was detected by sagittal images of MRCA, and common iliac artery aneurysm by coronal images of the renal artery.

## Conclusions

Asymptomatic atherosclerosis lesions including the coronary artery were detected by non-contrast MRA in 5% of participants. Non-contrast MRA is a useful and non-invasive technique without radiation exposure for vascular screening.

## Funding

None.

